# Exercise-induced microRNAs: molecular pathways and adaptive remodeling of skeletal muscle

**DOI:** 10.1007/s00424-026-03177-w

**Published:** 2026-05-09

**Authors:** Kübra Özdemir, Yeliz Demir

**Affiliations:** 1 Physical Education and Sports Teaching Department, Faculty of Sports Sciences, Atatürk University, Erzurum, 25030 Türkiye; 2 Department of Pharmacy Services, Nihat Delibalta Göle Vocational High School, Ardahan University, Ardahan, 75700 Türkiye; 3 Department of Chemistry, Faculty of Science, Atatürk University, Erzurum, 25240 Türkiye

**Keywords:** microRNAs, Exercise adaptation, Mitochondrial biogenesis, Exercise-induced signaling, Exosomal communication

## Abstract

Exercise is an effective physiological stimulus that promotes vast structural and functional changes in skeletal muscular tissue. Although transcriptional pathways that control exercise-based plasticity have been studied in depth, recent findings highlight the importance of microRNAs (miRNAs) as an essential post-transcriptional control platform mediating contractile stimuli and molecular and physiologic adaptations. This review provides a synthesis of existing evidence on exercise responsive miRNAs at the systems level, and especially on the topic of skeletal muscle remodeling. The review outlines the effects of different exercise regimens such as endurance, resistance, and high-intensity interval training to dynamic miRNA response to regulate major biological pathways such as mitochondrial biogenesis, protein turnover, angiogenesis, inflammatory signaling, and metabolic regulation. Acute exercise is also typified by temporary changes in miRNAs, which facilitate short-term stress signalling, and chronic training leads to more long-term miRNA re-programming, which facilitates long-term structural and functional remodeling. Also, circulating and exosome-bound miRNAs are also mentioned as potential agents of the muscle-to-organ interaction, thus supporting the idea of skeletal muscle that acts like an endocrine-like organ during exercise. Besides narrative synthesis, the review is based on an exploratory, data-driven re-analysis of publicly available skeletal muscle miRNA sequencing data to define convergent regulatory programs instead of data-specific differentiation. This is observed under integrative pathway and network-level analysis which identifies coordinated miRNA modules linked to mitochondrial regulation, cytoskeletal remodeling, and inflammatory control. Taken together, this framework brings together non-homogenous evidence throughout the literature and highlights the prospect of exercise-sensitive miRNAs as systems-level regulators of skeletal muscle adaptation.

## Introduction

Physical exercise has been generally recognized as one of the most effective biological stimuli that can be used to promote human health and life span (Özdemir and Demir 2025). Aerobic endurance training, resistance exercise, high-intensity interval sessions, long-term low-intensity exercise and the varied exercise modalities have deep and far-reaching responses in the skeletal muscle, cardiovascular, metabolism, nervous, and immune systems (Wu et al. 2023; Mohammed et al. 2025). These mechanisms do not limit to macroscopic physiological transformations but instead involve a massive reorganization of the molecules and cellular activities (Ellison et al. 2012). The collective activation of vast array of intracellular pathways is caused by mechanical loading, increased energy requirement, calcium signalling variations, reactive oxygen species (ROS), and responses to hormones (Attwaters and Hughes 2022). Therefore, exercise alters mitochondrial structure and activity, increases capillary density, shifts muscle fiber types, increases glucose and lipid metabolism, is linked to protein synthesis and breakdown and is also regenerative (Smith et al. 2023; Mølmen et al. 2025). Exercise in this case is not a mere physiological stressor but a holistic controller reestablishing homeostatic is linked to and enhancing the capacity to adjust to a stressor (Travers et al. 2022).

The problem of the regulation of such complex and coordinated adaptations at the molecular level has long been a major topic of interest of exercise physiology. Despite the many transcriptional and translational modulations that occur due to exercise being reported, the post-transcriptional regulatory layer has been receiving a growing interest over the past few years (Vita et al. 2022; Williams et al. 2025). Of these regulatory factors, microRNAs (miRNAs) have become key factors influencing exercise-induced tissue remodeling (Qin et al. 2025). miRNAs are short, non-coding, and small RNA molecules of about 20—24 nucleotides, which regulate the gene expression through stimulating mRNA degradation or inhibiting translation (George et al. 2024). One miRNA is capable of regulating hundreds of genes, and even an individual gene can be regulated by several miRNAs, thus, creating highly complex and tightly controlled gene networks.

The expression of a specific repertoire of miRNAs in skeletal muscle, so-called myomiRs (e.g., miR1, miR133a/b, miR206), are required to mediate myogenesis, fibre-type differentiation, satellite cell activation, regeneration and preservation of contractile properties (Pircher et al. 2021). It has been demonstrated that exercise can modify the expression of these miRNAs which are resident of muscle, indicating that they are parts of the adaptive response (Egan and Sharples 2023). In addition to their role in the cellular environment, miRNAs may also be extracellularly released, in exosomes, microvesicles, or protein complexes, and thus they have a systemic messaging effect (Makarova et al. 2021). Bouts of acute exercise result in more immediate fluctuations of the circulating miRNA concentration, but chronic training produces more consistent changes, some of which are linked to aerobic capacity, training status, and physiological adaptation (Witvrouwen et al. 2021). In this respect, miRNAs are in a strategic position of local tissue remodelling and systemic communication, acting as both mechanistic regulators and exercise responses biomarkers (Sanchis-Gomar et al. 2021).

The existing literature is still disjointed and inconsistent even though significant strides have been made. The research has shown outstanding heterogeneity in terms of exercise modality and intensity (Huang et al. 2023), duration time of sample collection (Sjöros et al. 2021), characteristics of participants (Gaemelke et al. 2022), and the platform of the analytic tools such as quantitative PCR, microarray (Feng et al. 2022), and next-generation sequencing (Fernandez‐Sanjurjo et al. 2024). In addition, the type of samples used in studies is diverse, i.e., skeletal muscle biopsies, serum, plasma, or isolated exosomal fractions can be used (Bizjak et al. 2021; Newmire and Willoughby 2022). These differences in methodology inhibit the capability of the field to find standard, repeatable miRNA signatures that are trustworthy in reacting to exercise (Dos Santos et al. 2022). As a result, a coherent picture of miRNAs emerged as effective indicators of training adaptation or a mechanism to which miRNAs are systematically regulated has not been formed.

The other significant gap in the literature is a lack of mechanistic clarity. Although many studies have observed a response in certain miRNAs after exercise, few studies have associated these miRNAs with validated or predicted target gene and those which do tend to integrate targets into coherent biological pathways (Masi et al. 2016; Li et al. 2025). Thus, the role of miRNAs responsive to exercise in mitochondrial biogenesis, protein turnover, angiogenesis, inflammatory signaling, or oxidative stress regulation is still underdeveloped. This mechanistic gap also constrains the translational usefulness of miRNAs in prescription of exercise, training load, rehabilitation or individualized exercise medicine.

Another weakness is the under exploitation of big publicly available data. Various miRNA sequencing datasets that were produced by pre- and post-exercise skeletal muscle or blood samples are available in repositories like the Gene Expression Omnibus (GEO) (D’Souza et al. 2017; Pan et al. 2025). Nonetheless, a majority of the reviews present a summary of previous findings without any analyses, which leaves the field without standardized computational methods that might give more insight into the clustering patterns, different miRNA expression signal patterns, or commitment to particular biological pathways. Such a bioinformatics-enhanced re-analysis of review studies could significantly enhance the level of mechanistic understanding, but this is not a common practice in the existing literature.

Considering such constraints, the combination of a systematic, mechanistically inclined, and data-driven synthesis of miRNA roles in exercise adaptation is urgently required. The current review hopes to address this gap by giving a synthesized review on miRNAs that are responsive to acute and chronic exercise both in skeletal muscle and circulating compartments. Besides reviewing the existing evidence, the review analyzes the molecular pathways that might be controlled by exercise-responsive miRNAs, and this analysis focuses on the axes of signalling known to mediate muscle plasticity (PGC-1α-medicated mitochondrial biogenesis), to hypertrophy (PI3K/Akt/mTOR), to protein breakdown and synthesis (FOXO), to angiogenesis (HIF -1 α-mediated), and to inflammatory responses (NF-κB).

Although the evidence on exercise-induced miRNA responses is growing very fast, the literature still lacks a substantial amount of homogeneity and conceptual fragmentation. Altered miRNA measurements have often been reported to be dependent on the exercise modality, intensity, duration, time of sampling, tissue origin, and characteristics of the participants giving inconsistent and even conflicting results (Cui et al. 2016; Horak et al. 2018). As an example, a number of miRNAs such as miR206, miR23a, miR486, among others have been reported to follow divergent expression patterns in acute and chronic exercise paradigms (Li et al. 2020; Li et al. 2025). It is therefore yet unknown whether miRNA alterations that are being observed are isolated regulations or part of coordinated adaptive programmes that regulate skeletal muscle plasticity. Further, the majority of the current research is based on descriptive profiling of individual miRNAs, but little is combined to form coherent biological pathways, which limits the interpretative capabilities of mechanistic significance and application to translation.

To solve these drawbacks, the current review attempts to deliver an integrative and systems-level synthesis of exercise-responsive miRNAs in skeletal muscle. Besides an overview of evidence of acute and long-term exercise research, the research also includes exploratory and data-driven re-analysis of publicly available skeletal muscle miRNA sequencing data to reveal common regulatory dynamics, as opposed to dataset-specific differentiation. Through the integration of literature-based mechanistic knowledge with pathway enrichment, target overlap evaluation and expression-based clustering studies, the review aims at placing single miRNA results into the framework of larger regulatory roles in exercise-mediated skeletal muscle remodelling. This is an integrative model that is supposed to be used to resolve the disparate observations in the literature and to build up on the knowledge of the miRNAs as coordinated post-transcriptional regulators connecting the exercise stimuli with the structural and functional remodeling in the long term. Throughout this review, the term “regulation” is used in a broad conceptual sense to describe molecular associations reported in the literature and does not necessarily imply direct causal mechanisms unless experimentally validated.

Rather than presenting a purely descriptive summary of exercise-responsive miRNAs, this review aims to provide a conceptual synthesis of the literature by proposing a network-oriented, systems-level perspective on miRNA regulation during exercise adaptation. By integrating findings from diverse experimental contexts, this framework highlights how multiple miRNAs may converge on shared regulatory pathways and coordinated molecular networks. Such a systems-level perspective may help organize heterogeneous observations reported across studies and provide a conceptual basis for future investigations exploring the complex regulatory architecture of exercise-induced skeletal muscle adaptation.

## Biogenesis and functional roles of miRNAs in skeletal muscle

miRNAs are small, non-coding RNA molecules that regulate gene expression at the post-transcriptional level and play an essential role in maintaining skeletal muscle homeostasis and plasticity (Chavez-Guevara et al. 2025). They are generated through a conserved biogenesis pathway involving the sequential processing of primary miRNA transcripts by the Drosha–DGCR8 complex in the nucleus and Dicer in the cytoplasm, ultimately yielding mature miRNAs that are incorporated into the RNA-induced silencing complex (RISC) (Pong and Gullerova 2018). Within this complex, miRNAs modulate gene expression primarily by repressing translation or promoting the degradation of target mRNAs (Huntzinger and Izaurralde 2011).

Importantly, accumulating evidence indicates that miRNA biogenesis and activity in skeletal muscle are not static but dynamically regulated in response to physiological stimuli such as mechanical loading, metabolic stress, calcium flux, and redox signaling, all of which are hallmarks of exercise (Kirby and McCarthy 2013; Diniz and Wang 2016). Exercise-induced alterations in miRNA processing efficiency, stability, and intracellular localization suggest that miRNA regulation constitutes an adaptive layer that fine-tunes gene expression in accordance with contractile and metabolic demands (Domańska-Senderowska et al. 2019). Accordingly, miRNAs should be viewed not merely as constitutive regulators of muscle gene expression but as responsive modulators that contribute to both acute exercise signaling and long-term training-induced remodeling (Masi et al. 2016).

### Muscle-enriched miRNAs (MyomiRs) in skeletal muscle regulation

A set of myomiRs, miR-1, miR-133a, miR-133b, miR-206, miR-208b, miR-486, and miR-499 are expressed by skeletal muscle (Chen et al. 2006; Zilahi et al. 2019). These miRNAs are either myogenic-specific, or they are highly expressed in muscle tissue and are regulated by master myogenic factors, such as MyoD1, Myogenin, myocyte enhancer factor 2C (MEF2C), and serum response factor (SRF) (Aránega et al. 2021). The myomiRs act as central regulators of myogenic differentiation, myofiber type specification, excitation contraction coupling and tissue regeneration (Zhelankin et al. 2023).

Functionally, myomiRs play complementary roles in balancing myoblast proliferation, differentiation, and maintenance of mature muscle fibers (Güller and Russel 2010). miR-1 and miR-206 are predominantly associated with the promotion of myogenic differentiation, acting to reinforce terminal myogenic programs and stabilize muscle-specific gene expression (Kim et al. 2006). In contrast, miR-133a and miR-133b are linked to proliferative states, supporting the expansion and maintenance of myogenic precursor populations. Through this functional partitioning, myomiRs establish a regulatory equilibrium between growth, differentiation, and tissue stability (Cui et al. 2019).

Beyond myogenesis, specific myomiRs contribute to the preservation of muscle fiber identity. miR-208b and miR-499, which are encoded within intronic regions of myosin heavy chain genes, are key determinants of fiber-type characteristics. Their expression aligns with slow-twitch muscle gene programs and supports the maintenance of contractile and metabolic specialization across muscle fiber populations (Zhelankin et al. 2023). Collectively, these myomiRs form an interconnected regulatory circuit that defines the structural and functional phenotype of skeletal muscle at baseline.

### Regulation of myogenesis and satellite cell function by miRNAs

The ability of skeletal muscle to regenerate through myogenesis is attributed to the presence of a pool of resident stem cells, known as satellite cells (Dumont et al. 2015). miRNAs mediate widespread regulation of the various functions such as activation, proliferation, differentiation and self-renewal of the satellite cells (Fochi et al. 2020). Several miRNAs have been identified as key regulators of satellite cell behavior. miR-489 is associated with the maintenance of quiescence, contributing to the long-term preservation of the satellite cell pool (Patel et al. 2019). In contrast, miR-195 and miR-497 participate in the regulation of cell-cycle progression, influencing the proliferative capacity of activated satellite cells (Qiu et al. 2017). During the differentiation phase, miR-1 and miR-206 facilitate the transition toward mature myogenic phenotypes by reinforcing differentiation-associated gene expression programs and suppressing progenitor-state markers (Goljanek-Whysall et al. 2011).

MiRNA-mediated regulation of satellite cells does not operate in isolation but is tightly coordinated with canonical myogenic transcription factors. This coordinated control ensures that regenerative processes proceed in a temporally ordered manner, preventing premature differentiation or excessive proliferation (Boutet et al. 2012). Through these mechanisms, miRNAs serve as molecular gatekeepers that stabilize muscle regeneration while preserving the long-term regenerative reserve of skeletal muscle.

### miRNAs in muscle fiber -type specification and contractile identity

There are significant distinctions in skeletal muscle fibers metabolism and contractile characteristics. Type I muscle fibers predominantly rely on oxidative metabolism, whereas type II fibers exhibit a greater dependence on glycolytic pathways. miRNAs play an active regulatory role in establishing and maintaining these distinct metabolic and contractile phenotypes (Wang et al. 2024). Among the miRNAs implicated in fiber-type regulation, miR-499 and miR-208b occupy a central position. These miRNAs promote the expression of gene programs associated with slow-twitch muscle fibers by suppressing transcriptional repressors of oxidative muscle genes. Their sustained expression contributes to the stabilization of fiber-type identity and reinforces contractile specialization within muscle tissue (Liu et al. 2016).

### miRNA-mediated control of metabolic homeostasis and mitochondrial regulation

Mitochondria are the core mediators of skeletal-muscle adaptation, and miRNAs play an imperative role in regulating mitochondrial biogenesis, proliferation, and oxidative capability (Lima et al. 2017); in turn, mitochondrial biogenesis is regulated by down-regulation of miR-23a by exercise (Krammer et al. 2022) and mitochondrial fission and oxidative capacity by miR-494 (Pálešová et al. 2025). Besides, miR-378 and miR-29 families coordinate lipid oxidation and glucose metabolism (Agbu and Carthew 2021). Since exercise presents a high metabolic load, metabolic and contractile stimuli-responsiveness of these miRNAs allows quick and effective reorganization of cellular energy networks. This regulatory framework explains why even a single exercise session is capable of triggering quantifiable molecular changes that allows skeletal muscle to be pre-coded to an increased metabolic burden (Grieb et al. 2023).

### miRNAs in the regulation of protein synthesis, degradation, and muscle mass maintenance

The maintenance of skeletal muscle mass depends on a finely tuned balance between anabolic and catabolic processes (McCarthy and Murach 2019). MiRNAs participate in the regulation of this balance by modulating pathways associated with protein synthesis, proteolysis, and muscle integrity (Francisco et al. 2022). miR-486 is commonly linked to anabolic signaling environments, supporting mechanisms that favor protein accretion and muscle mass preservation (Liu and Dong 2025). In contrast, miR-29 family members are associated with regulatory contexts that promote protein degradation and muscle atrophy when dysregulated (Li et al. 2017). Additionally, miR-206 has been implicated in the modulation of proteolytic systems, contributing to the control of muscle protein turnover. By integrating signals that influence both synthesis and degradation, miRNAs function as stabilizers of muscle mass under homeostatic conditions (Ma et al. 2015). Their coordinated activity ensures that skeletal muscle maintains structural integrity while retaining the capacity for growth or remodeling when required. Together, these findings establish miRNAs as central regulators of skeletal muscle biology, governing myogenesis, fiber identity, metabolic balance, and tissue maintenance under basal conditions. Understanding how these regulatory systems are dynamically modulated by physiological stimuli requires examination of exercise-induced temporal changes in miRNA expression, which is addressed in the following section.

## Exercise-induced regulation of miRNAs: acute and chronic adaptations

Skeletal muscle is subjected to a plethora of mechanical, metabolic, hormonal and neural stimuli by exercise, which trigger particular intracellular signalings, reorganizing the transcriptional and post-transcriptional landscape (Zheng et al. 2025). Of these regulatory layers, miRNA has become particularly important as an intermediate of muscle plasticity that can control intricate mitochondrial biogenesis and angiogenesis pathways, hypertrophy, inflammation, and whole system metabolic regulation. The dynamics of miRNAs in relation to exercise of various types is critical in explaining the training adaptation at a molecular level (Marceca et al. 2020). This part is the summary of the existing evidence of the acute and chronic exercise-induced miRNA regulation and their possible use as biomarkers of physiological stress and adaptation.

In acute exercise, which is considered to be a single episode of endurance, resistance or high-intensity exercise, miRNA content changes in the skeletal muscle and in circulation are rapid, but short-lived (Archer and Lindahl 2019). These changes indicate the direct signalling requirements on contracting fibres. Some myomiRs are especially acute mechanical loading sensitive including miR1, miR133a/b and miR206. The increase in circulating concentrations of miR-1 and miR-133 in most studies following strenuous exercise is significant and probably due to the regulated export, but not passive leakage (Li et al. 2013). Meanwhile, miR-206 concentration in muscle tends to be raised to contribute to the early regenerative and differentiation through regulating the activity of Pax7 and HDAC4 (Srivastava et al. 2021; Chen et al. 2024). Other miRNAs, including miR 486, typically fall out in the hours after an acute session, and release the inhibition of PTEN, allowing transient activation of the Akt pathway (Qiu et al. 2024). miR-208b and miR-499, in their turn, orchestrate myofiber type identity by inhibiting transcriptional repressors of slow-twitch muscle genes, thereby favoring a slow-oxidative phenotype (Li et al. 2022).Together, the myomiRs form a complex regulatory circuit that coordinates myofiber development, preservation and adaptive remodelling. A significant part of the plasticity that is expressed in skeletal muscle during exercise training is due to their sensitivity to neuromuscular, biomechanical, and metabolic stimuli (Giagnorio et al. 2021).

Acute miRNA responses are also influenced by metabolic stress. Long-duration aerobic exercise and moderate-to-high intensity exercise suppress the expression of several miRNAs that typically repress oxidative metabolism, including miR-23a, miR-494, and miR-696, particularly in the post-exercise period (Chavez-Guevara et al. 2025). This downregulation is associated with enhanced PGC-1α–mediated mitochondrial biogenesis, a key mechanism underlying exercise-induced metabolic adaptation (Krammer et al. 2022). On the contrary, miRNAs like miR-23a and miR-27a control signalling cascades, which control the expression of glycolytic genes. These miRNA-regulatory networks are linked closely to exercise-induced changes in fiber-type composition (Wang et al. 2017). Whereas resistance training is associated with miRNAs associated with fast-twitch hypertrophy, endurance training is associated with miRNAs that promote oxidative phenotypes thus facilitating mitochondrial biogenesis, as well as increasing fatigue resistance (Kolodziej et al. 2022). Likewise, ROS-vectored pathways are able to alter miRNA expression, in which miR-21, miR 146a and miR 200 family can respond to temporary oxidative stress. These acute oscillations finely tune the relationship between inflammatory signalling and antioxidative defence, in such a way that muscle does not enter a maladaptive inflammatory response.

The circulating miRNAs compose a significant portion of acute response. An example of high-intensity interval exercise is shown to elevate miR -1, miR -133a, miR -206 and miR -222, both of which are muscle-derived signals and systemic cardiovascular-stress signals (Magenta et al. 2016). Also, these miRNAs are associated with lactate buildup, heart rate and perceived exertion indicating their potential application as biomarkers of monitoring external and internal load. Notably, acute responses normally subside in 24 h hence their short-term signalling properties but not long-term molecular remodelling (Baggish et al. 2014). Consequently, circulating miRNAs are increasingly recognized as sensitive indicators of acute physiological load, bridging the gap between exercise-induced mechanical stress and systemic recovery dynamics.

Prolonged exercise training prompts more consistent and still lasting alterations in miRNA expression which is the indication of structural and functional remodelling of skeletal muscle (Ultimo et al. 2018). Regular endurance exercise causes sustained suppression of miRNAs which suppress mitochondrial growth and oxidative metabolism (Da Silva et al. 2020). Long-term miRNA regulation is an additional form of angiogenic adaptation; chronic endurance training elevates basal levels of the miRNAs miR -126 and miR -210, both of which induce a capillarization response and endothelial repair by regulating the VEGF and hypoxia-responsive pathways (Rusek 2025). Another response is the appearance of anti-inflammatory changes during chronic exercise. Repeated up-regulation of miR-21 and miR-146a suppresses the extravagant inflammatory signalling and increases the muscle resilience to repeated mechanical loads. The changes are in line with the long-established anti-inflammatory phenotype in trained individuals. Also endurance training alters miRNAs that mediate substrate utilisation and insulin sensitivity implying that miRNAs help in enhancing metabolic health following long-term training (Niu et al. 2025).

The miRNA profile induced by resistance training is also unique and linked to hypertrophy and protein turnover. Hypertrophy is also linked to decreased expression of miR-29 and other miRNAs which inhibit IGF-1 signalling or enhance protein degradation (Habibi et al. 2016). Unlike endurance training, miR-206 can reduce on a long-term basis during hypertrophy, which restricts differentiation cues and enhances fibre growth over repair (Isanejad et al. 2016). Hybrid miRNA responses are realized by high-intensity interval training, which combines both mechanical and metabolic stress. High-intensity interval training (HIIT) enhances angiogenic miRNAs like miR-21 and miR-126 and at the same time inhibits metabolic inhibitors such as miR-23a and miR-696 (Lamon and Russell, 2017). HIIT has been especially remarkable with respect to a potent impact on the circulating miRNAs that relate to the cardiovascular adjustment process; miR-21, miR-222, and miR-126 have been associated with the enhancement of endothelial functionality (Horak et al. 2018).

Sex, age and training status mediate the responsiveness of miRNAs to exercise. An example is estrogen, which alters the activity of Dicer and influences the expression of a number of muscle-related miRNAs, causing sexual variations in baseline levels, as well as responses to exercise (Hiam et al. 2023). Older adults have a decreased plasticity in miRNA network, which adds to the dulling of molecular responses and anabolic resistance (Margolis and Rivas 2018). The acute reaction of trained individuals is frequently attenuated in comparison to the untrained individuals, apparently because the processes of transcription as well as metabolism are more constant. A notable aspect of miRNA regulation in response to exercise is inter-tissue communication through exosomes. Signalling muscle-to-liver, muscle-to-heart, and muscle-to-adipose Contracting muscle releases miRNA-rich exosomes into circulation, which allow signalling between muscle and liver, muscle and heart, and muscle and adipose (Csala et al. 2025).

On the whole, exercise elicits multifaceted and well-coordinated miRNA-responses falling within the spectrum of both an acute signalling and chronic remodelling. Acute variations act as transient regulators of metabolic, inflammatory and regenerative programs and chronic adjustments maintain miRNA designs that facilitate augmented oxidative ability, hypertrophy, angiogenesis and metabolic efficiency. The results highlight the promise of miRNAs as training load, adaptation and performance biomarkers and also emphasise their future use as therapeutic targets in diseases with the identifyable characteristics of disrupted muscle performance or metabolic regulation.

## Molecular pathways and systems-level integration of exercise-responsive miRNAs

The evidence summarized in the previous section demonstrates that both acute and chronic exercise induce distinct and reproducible alterations in skeletal muscle and circulating miRNA profiles. While these observations clearly establish miRNAs as exercise-responsive molecules, their biological relevance becomes apparent only when these expression changes are interpreted within the context of intracellular signaling pathways and systems-level adaptation. In this regard, miRNA should not be viewed as isolated molecular switches but rather as integrative regulators that coordinate multiple signaling axes governing skeletal muscle plasticity.

**Fig. 1:**
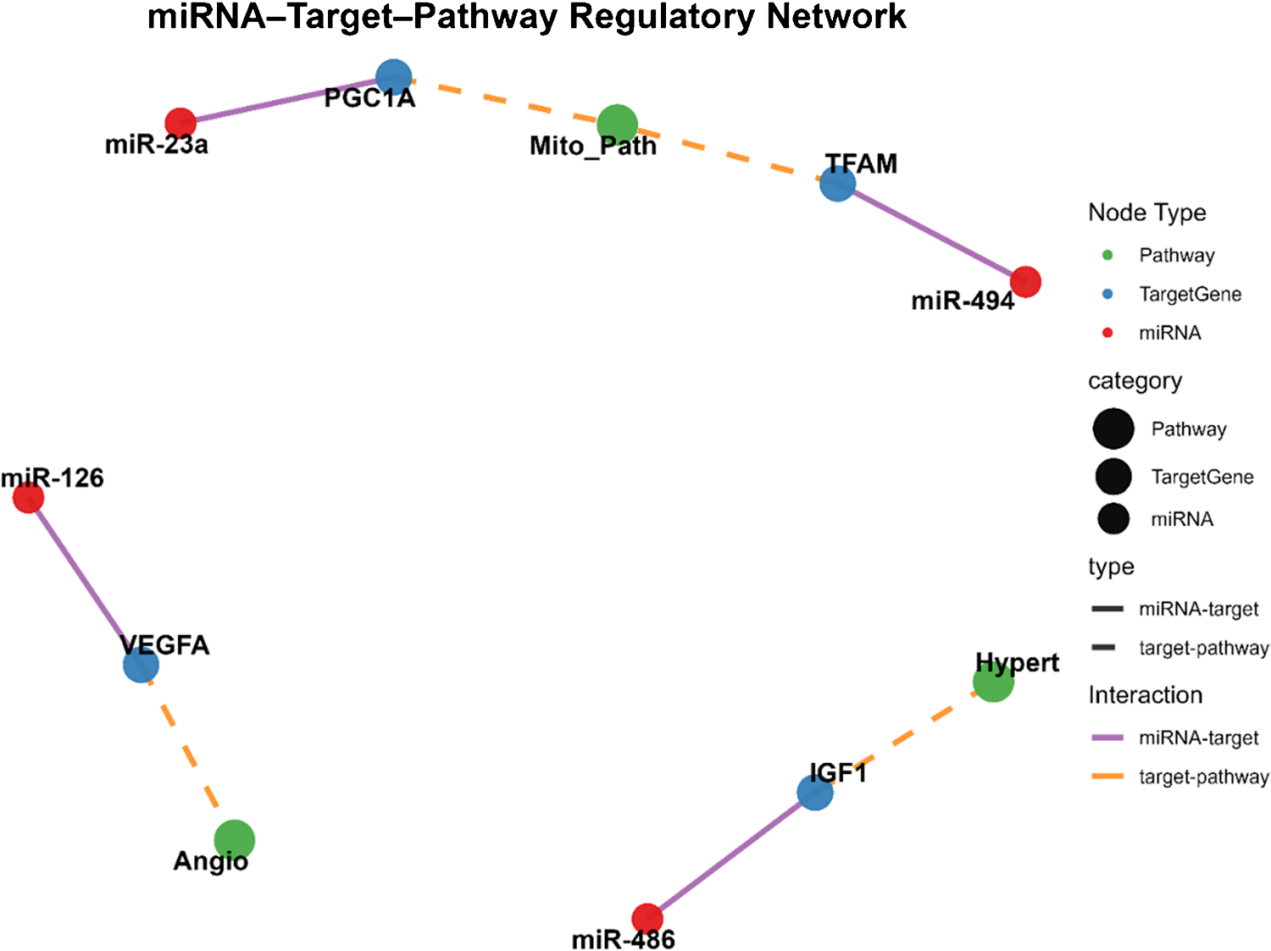
Network-based representation of exercise-responsive miRNA interactions and associated biological pathways

One of the most consistently reported targets of exercise-responsive miRNAs is mitochondrial biogenesis and oxidative metabolism. Endurance-type exercise suppresses several miRNAs known to negatively regulate mitochondrial regulators, thereby facilitating the activation of the PGC-1α transcriptional program. miRNAs such as miR-23a, miR-494, and miR-696 have been implicated in the repression of genes involved in mitochondrial proliferation and oxidative phosphorylation. As illustrated in Fig. 1, exercise-responsive miRNA may be conceptualized as components of interconnected regulatory networks in which multiple miRNAs converge on shared biological pathways involved in skeletal muscle adaptation. Their downregulation following acute endurance exercise, and sustained suppression with chronic training, removes post-transcriptional constraints on mitochondrial expansion. This regulatory layer provides a mechanistic explanation for how repeated contractile and metabolic stimuli translate into long-term improvements in oxidative capacity and fatigue resistance, even in the absence of sustained transcriptional activation (Chavez-Guevara et al. 2025).

Following muscle injury or exercise-induced damage, satellite cells exit their quiescent state and undergo a tightly regulated sequence of activation, proliferation, and subsequent differentiation into mature myofibers. This coordinated regenerative process is critically modulated by myogenic miRNAs, including miR-1, miR-206, and miR-133 (Chalchat et al. 2023). Differentiation is promoted by the suppression of Pax7 by miR1/206 (Falcone et al. 2014), and Myostatin modulated by miR-27 is a major negative muscle mass regulator (Wang et al. 2020). The crosstalk between satellite cell–specific miRNAs and canonical myogenic transcription factors ensures precise temporal coordination of muscle repair (Bianchi et al. 2017). Chronic exercise, particularly resistance exercise, is associated with the activity of these miRNAs, which may increase the regenerative potential of exercised animals (Davidsen et al. 2011).

The processes of muscle hypertrophy and atrophy result in a fine balance that keeps both anabolic and catabolic processes; miRNAs mediate both of these states (Margolis and Rivas 2018). miR-486 can control hypertrophy by inhibiting PTEN and activating Akt signalling (Shen et al. 2019), whereas miR-499 can control mTORC1 stimulation (Xie et al. 2020). On the other hand, miR-29 causes atrophy through the decreasing of IGF-1 and PI3K components, with miR-206 contributing to the suppression of proteolytic pathways. Changes in muscle protein turnover are closely linked to exercise-induced modulation of these miRNAs (Zhang et al. 2025).Exercise-induced remodeling generally shifts the miRNA expression profile toward anabolic signaling, whereas prolonged inactivity or pathological conditions promote miRNA signatures associated with muscle atrophy. It is critical to understand this regulatory layer to better understand interindividual differences in hypertrophic or strength responses to adaptation. This coordinated miRNAs reprogramming suggests that exercise-induced muscle growth is not solely dependent on upstream hormonal or mechanical cues but is critically shaped by post-transcriptional fine-tuning of anabolic–catabolic balance.

The network illustrates potential relationships between exercise-responsive miRNAs and key biological pathways involved in skeletal muscle adaptation. Nodes represent either mRNAs or biological pathways, while edges (connections) indicate reported or predicted associations between specific miRNAs and the pathways they may influence. The network structure highlights the possibility that multiple exercise-responsive miRNAs converge on shared biological processes, suggesting coordinated regulatory behavior rather than isolated molecular effects. This conceptual representation aims to illustrate how heterogeneous observations reported across individual studies may be organized within a broader systems-level framework.

Beyond muscle-intrinsic adaptation, exercise-responsive circulating miRNAs reflect early systemic metabolic dysregulation. In prediabetic individuals, circulating miR-192 and miR-193b are selectively elevated, whereas their levels normalize in overt diabetes, indicating specificity to early glucose intolerance. Notably, this miRNA pattern is conserved across species and is similarly observed in glucose-intolerant mouse models. Chronic therapeutic exercise restores circulating miR-192 and miR-193b to baseline in parallel with metabolic normalization in both humans and mice (Párrizas et al. 2015). This dynamic reversibility highlights circulating miRNAs as integrative, exercise-sensitive regulators that link metabolic stress with systemic adaptation, reinforcing their potential role as early biomarkers and modulators of whole-body metabolic homeostasis.

MiRNA homeostasis provided by exercise also overlaps with inflammatory and redox sensitive signalling. It is known that regular physical activity leads to an anti-inflammatory phenotype, and miRNAs are an essential part of this process. As an example, miR-146a and miR-21 mediate NF-κB signalling and suppress excessive inflammatory reactions after repeated mechanical loading (Ultimo et al. 2018). At the same time, redox-sensitive miRNAs react to exercise-initiated changes in ROS, allowing adaptive and not maladaptive inflammatory signalling. This balance facilitates the prevention of chronic low-grade inflammation and maintenance of acute inflammatory cues required to facilitate tissue repair and remodelling.

The functions of exercise-responsive miRNAs are not limited to the intracellular ambiance of skeletal muscle at the systems level. The release by contraction of miRNA-enriched extracellular vesicles into the circulation aids in communication with other body organs, including liver, heart and adipose tissue. MiRNAs exosomaled miR-1, miR-133, and miR-486 have been found to be involved in the regulation of hepatic glucose metabolism, cardiac remodeling, and the functioning of adipocytes (Aoi et al. 2013; Fathi et al. 2020). The new paradigm makes skeletal muscle an endocrine organ, where miRNAs are hormone-like messengers that co-ordinate the whole-body response to exercise.

Integratively, publicly available sequencing datasets also provide support to the fact that exercise yields coordinated mRNA-network rather than individual expression changes. Re-examination of skeletal muscle miRNA data show patterns of clustering that can be explained by mitochondrial control, angiogenesis and protein turnover, which supports the idea of miRNAs as nodal regulators of exercise-induced remodeling. Even though data-driven methods have not been exploited in the literature, they give a robust platform of finding consistent regulatory signatures across exercise modes and groups.

With these results combined, it can be emphasized that miRNAs are placed at the heart of the matter of transducing mechanical, metabolic, and hormonal stimuli of exercise into an organized molecular and physiological adaptation. Through their simultaneous coordination of numerous signalling pathways, and their ability to induce inter-tissue communication, exercise-responsive miRNAs provide a common regulatory layer that bridges acute signalling events with chronic structural and functional remodelling. The fact that they are incorporated at the systems-level underscores their potential also as biomarkers of training adaptation, as well as, targets to personalised exercise prescription and rehabilitation interventions.

## Data-driven integrative analysis of exercise-responsive miRNA networks

Many studies have reported changes in individual miRNAs in response to exercise (Xu et al. 2015; Domańska-Senderowska et al. 2019), but the extent to which these molecules converge on shared regulatory programs remains insufficiently explored. To complement the narrative synthesis presented in this review, an exploratory integrative analysis was conducted focusing on a curated set of exercise-responsive miRNAs that have been consistently reported across endurance, resistance, and high-intensity exercise studies. Based on literature consensus, a core group of miRNAs (hsa-miR-1—3p, hsa-miR-133a-3p, hsa-miR-133b, hsa-miR-206, hsa-miR-486—5p, hsa-miR-23a-3p, hsa-miR-378a-3p, hsa-miR-21—5p, and hsa-miR-146a-5p) was selected for integrative analysis. Experimentally validated gene targets were compiled from curated databases and literature sources and subsequently subjected to Reactome pathway enrichment analysis to identify biological processes potentially coordinated by these miRNAs. In addition, target overlap analysis and hierarchical clustering using publicly available skeletal muscle miRNA expression data (GEO dataset GSE127187) were used to explore potential regulatory convergence and coordinated expression dynamics. Importantly, this integrative analysis was designed to illustrate systems-level regulatory patterns rather than to generate dataset-specific differential expression claims.

**Fig. 2:**
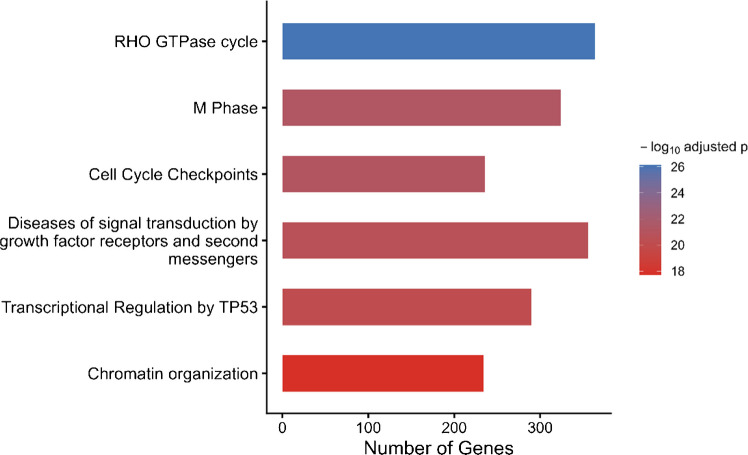
Reactome pathway enrichment analysis of validated gene targets of core exercise-responsive miRNAs. Bar length represents the number of genes associated with each pathway, and color intensity reflects − log_10_ adjusted p-values

An examination of pathways revealed a significant overlap of miRNAs responsive to exercise at the core of regulation of cytoskeleton dynamics, cell-cycle regulation, and chromatin organization (Fig. 2). One of the most highly enriched pathways called the RHO GTPase cycle was the central node, thus underscoring the importance of the actin remodeling, mechanotransduction, and force transmission to skeletal-muscle adaptation to exercise. The simultaneous up-regulation of both cell-cycle checkpoints and M-phase-sensitive pathways also suggests that the exercise-responsive miRNAs combine mechanical and metabolic cues with cellular stress-detection and growth-regulatory pathways.

Among others, pathways relating to TP53-related transcriptional regulation and chromatin organization were highly enriched, which supports the hypothesis that exercise causes persistent epigenetic and transcriptional reprogramming. These findings are consistent with new findings that exercise may induce lasting molecular memory in skeletal muscle, thus having the potential to maintain adaptive capacity even during periods of detraining (Beiter et al. 2020). More importantly, the overlap of specific miRNAs on common regulatory axes indicates that adaptation to exercise is regulated by specific miRNAs networks but not by individual post-transcriptional events.

**Fig. 3:**
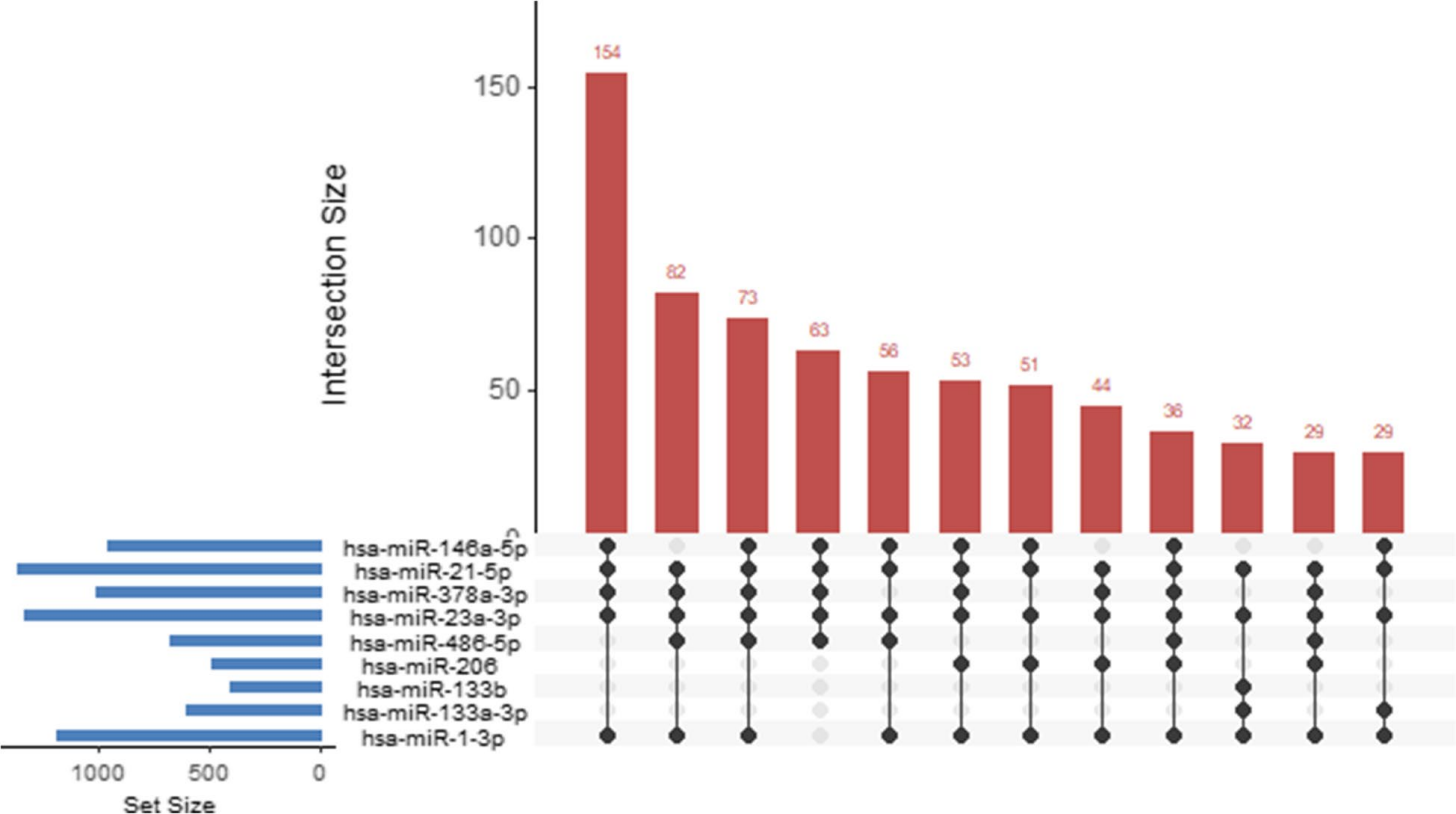
UpSet plot representing the intersection of the experimentally validated target genes of exercise responsive miRNAs. Gene analysis was restricted to those involved in enriched Reactome pathways and shared by at least five exercise responsive miRNAs, thus highlighting strong regulatory convergence. The size of the gene-intersections common to the different combinations of miRNAs is denoted by bars; the total number of targets of any miRNA is denoted by the horizontal bars. To be more specific, only the biggest crossroads are depicted. The figure illustrates a vast overlapping of canonical myomiRs and metabolically or inflammation-linked miRNAs, hence pointing out coordinated post-transcriptional regulation of underlying exercise-induced skeletal-muscle adaptations

To question the scale of post-transcriptional convergence amidst exercise-responsive miRNAs, an UpSet examination was performed, with a focus on those genes that are commonly targeted by a collection of miRNAs. The criteria applied to the analysis were to experimentally validate targets involved in enriched Reactome pathways, and to genes regulated simultaneously by at least five miRNA responsive to exercise, thus placing the emphasis on robust and biologically significant regulatory overlap. As shown in Fig. 3, there is a large set of genes shared among miRNA sets across divergence that reveals a highly convergent regulatory structure. Outstandingly, the biggest crossroads consist of canonical myomiRs and miRNAs associated with metabolism or inflammation, which suggests that the pathways related to cell-cycle regulation, signal transmission, and chromatin structure are coordinated (Russell et al. 2013; D’Souza et al. 2018). This overlap supports the idea that the adaptations that happen as a result of exercise are mediated by whole miRNA networks, and not by single miRNA modalities.

**Fig. 4:**
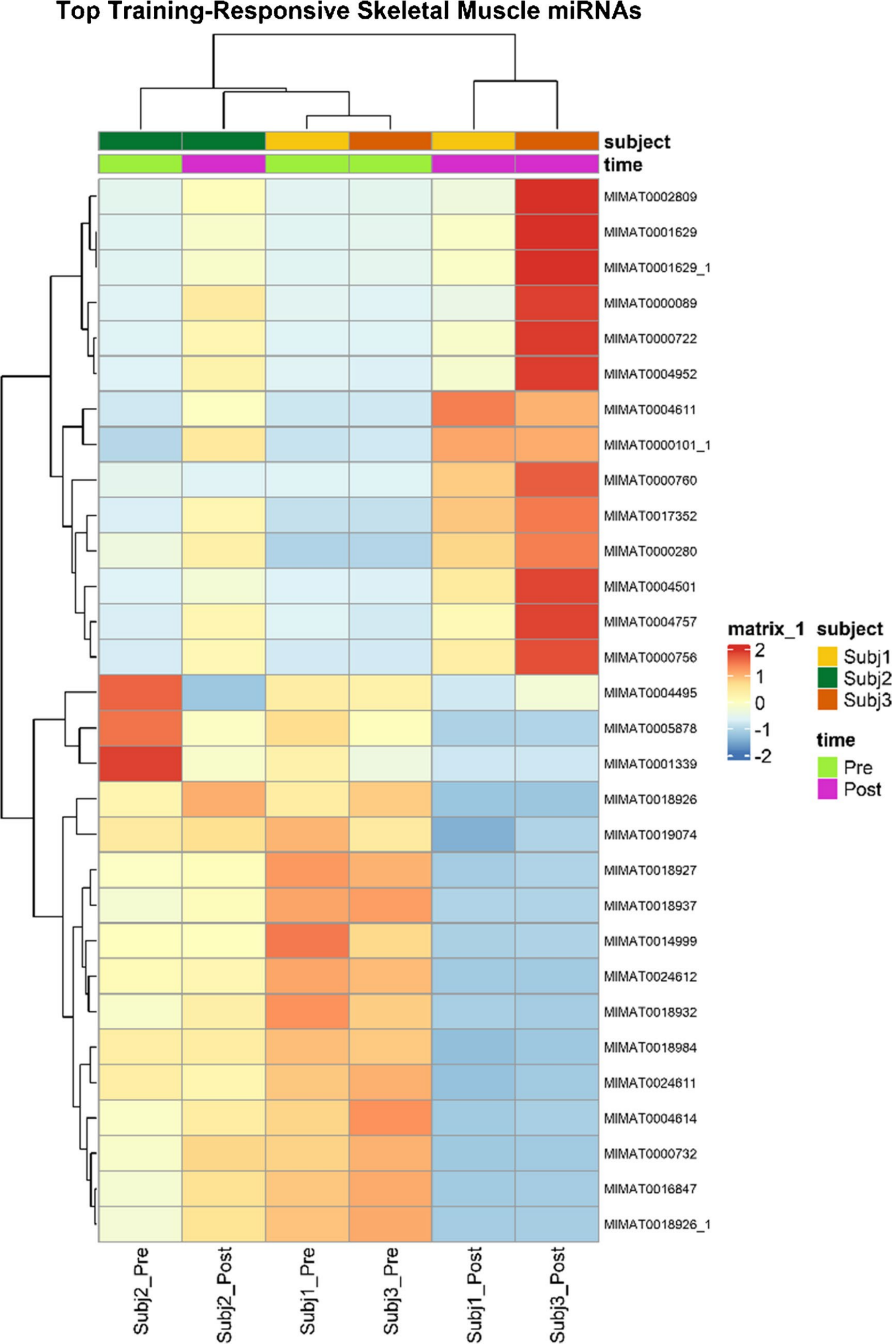
Exercise-responsive miRNAs were visualized as heatmap and hierarchical clustering of the expression data of the GEO dataset GSE127187. The heatmap presents a concerted expression patterns of chosen miRNAs under pre- and post-exercise statuses and different people. The intensity on color is normalised relative expression levels. The visualisation is meant to highlight co-regulatory behaviour and modular organisation of exercise-responsive miRNAs, as opposed to inferring dataset-specific differential expression

Hierarchical clustering and heatmap visualisation were used in order to define the coordinated expression dynamics of exercise-responsive miRNAs using publicly available expression data, retrieved as the GEO dataset GSE127187 (Massart et al. 2021). This comparison was supposed to provide a synthetical and representative picture of miRNA co-regulation, not to support dataset specific claims of differentiation. As in Fig. 4, exercise-sensitive miRNAs have coherent clustering behaviour, and therefore, their training transcriptional response is non-random and organized.

In a variety of topics, post-exercise samples are significantly different than pre-exercise ones, which suggests that exercise induces a uniform directional change in miRNA expression patterns. Significantly, the observed patterns are indicative of modular organisation, where subsets of miRNAs show common temporal dynamics among individuals. According to this observation, exercise-induced miRNA outcomes are regulated through a number of coordinated functional modules, as opposed to independent action of post-transcriptional events on single miRNAs.

Inter-subject variance is evident; however, the general organization of clustering is highly preserved across subjects, and it thus supports the idea that there is a common regulatory framework underlying exercise adaptation. Notably, the patterns of modular expression patterns as illustrated in the heatmap are closely related to the pathway-level convergence as indicated by Reactome enrichment analysis. miRNAs that exhibit a coordinated change in their expression also all converge on common biological pathways that mediate cytoskeletal remodelling, cell-cycle and chromatin organization. Enrichment of pathways, overlap of targets, and expression regulation analyses are combined and provide a network-scale perspective of miRNA-mediated regulation during exercise adaptation. Instead of acting alone, exercise-responsive miRNAs seem to form interdependent regulatory networks that centre on key molecular pathways that coordinate skeletal-muscle remodelling, cellular signalling and transcriptional reprogramming. This unifying view article brings together inconsistent results in the field as well as highlighting the importance of coordinated post-transcriptional regulation as a key means by which exercise induces adaptive responses at the molecular level. The framework facilitates the development of data-driven analysis strategies to drive the concept of miRNAs function out of individual association into an integrated systems-level framework.

## Translational ımplications, biomarker potential, and future directions

Growing evidence suggests that miRNA exercise responsive is a key regulatory interface where acute contractile and metabolic signals are coupled to long-term structural and functional skeletal muscle adaptation. These miRNAs are not isolated post-transcriptional modifiers, but instead integrative nodes, which coordinate a variety of signaling pathways that control mitochondrial functioning, protein turnover, angiogenesis, inflammation and inter-tissue communication (Egan and Sharples 2023). This integrative ability establishes miRNAs at the center of exercise-induced plasticity, between short-term molecular cues and long-term physiological rearrangements. The dynamic control of the circulating miRNAs has spawned much interest on their possible application as least invasive biomarkers of exercise load, state of training and adaptive capacity (McAdam et al. 2025). Changes in the index of aerobic fitness, muscular adapting and recovery have been linked to changes in the specific miRNA signatures after acute and chronic exercise (Domańska-Senderowska et al. 2019). However, the presence of significant methodological and biological heterogeneity is still one of the biggest obstacles to their translation. The differences in exercise modality, intensity, time points of sampling, biological matrices and analytical platforms have hindered the determination of strong and consistent miRNA signatures. In turn, as promising biomarkers of exercise responsiveness, circulating miRNAs should be carefully interpreted and highly validated in their clinical and performance-related application.

In addition to biomarker discovery, miRNAs that are responsive to exercise can be used to develop individual exercise medicine. Modulating miRNAs that regulate many important biological processes related to metabolic regulation, maintenance of muscle mass, vascular health, and inflammatory responses are potential sources of modifying exercise interventions in diverse populations. It is particularly applicable in aging, metabolic diseases and chronic disease conditions in which skeletal muscle plasticity is impaired. Combining miRNA responses and physiological and functional measurements may enable personalized exercise prescriptions aimed at maximizing adaptation and minimizing maladaptive responses. The standardized experimental designs and longitudinal studies should be given priority in future studies to ensure that the temporal dynamics of miRNA regulation in response to exercise is captured. Multi-omics data such as transcriptomics, proteomics, and metabolomics and sophisticated bioinformatics and network-based tools will be necessary to sketch out conserved regulatory modules of exercise adaptation. Moreover, one should focus more on sex-specific, age-specific, and training-status-related differences in miRNA responsiveness, as these variables have a severe impact on adaptive results. It is not only that by addressing these challenges we will improve the mechanistic understanding but will also be able to implement miRNA research to practical use in exercise science and rehabilitation.

To sum up, miRNAs represent a synthesizing regulatory system by which exercise may translate its multi-dimensional impacts on skeletal muscle and physiologic systems as a whole. The next step in realizing the full potential of descriptive profiling as a bio-marker and modulator of exercise-induced adaptation will be the development of integrative, mechanistic, and data-driven methods.

## Conclusion

Physical activity triggers intricate and highly orchestrated molecular responses within the skeletal muscle and it is slowly gaining momentum that miRNAs are at the center stage in coordinating these reactions. Nevertheless, most of the literature has treated exercise-response miRNAs in a disjointed fashion, with concise attention paid to respective molecules or specific pathways. This review combines both mechanistic and data-driven analytical perspectives of existing knowledge to synthesize the existing body of knowledge, and consequently offers an integrated system level view of miRNA-mediated regulation during exercise adaptation. During the combination of the pathway enrichment analysis, intersection-based target overlap evaluation, and expression-based clustering based on independent datasets, the current study proves that miRNAs that react to exercise do not act independently. Rather, they create regulatory networks that are connected, and all of them focus on a limited number of main biological pathways that control cellular plasticity, signal transduction, transcriptional regulation, and structural remodeling. The observed convergence between analytical layers suggests the possibility of coordinated post-transcriptional regulatory mechanisms underlying exercise-induced skeletal muscle adaptation. It is important to acknowledge that substantial heterogeneity remains across studies investigating exercise-responsive miRNAs. Variability may arise from multiple sources, including differences in exercise modality (endurance, resistance, or high-intensity interval training), timing of biological sampling following exercise, the specific tissue types analyzed (e.g., skeletal muscle, plasma, or circulating exosomes), and population characteristics such as training status, age, and sex. These methodological and biological differences may partly explain the variability observed in reported miRNA responses across studies. The integrative framework proposed in this review should therefore be viewed as a conceptual synthesis of existing evidence, aimed at organizing heterogeneous observations into a coherent systems-level perspective. While this approach does not resolve all sources of variability across studies, it highlights potential regulatory convergence among exercise-responsive miRNAs and provides a foundation for future experimental and multi-dataset investigations. This framework may help conceptualize the molecular interactions underlying exercise adaptation and provides a basis for future research.

## Data Availability

No datasets were generated or analysed during the current study.
